# Factors Influencing Engagement, Perceived Usefulness and Behavioral Mechanisms Associated with a Text Message Support Program

**DOI:** 10.1371/journal.pone.0163929

**Published:** 2016-10-14

**Authors:** Julie Redfern, Karla Santo, Genevieve Coorey, Jay Thakkar, Maree Hackett, Aravinda Thiagalingam, Clara K. Chow

**Affiliations:** 1 The George Institute for Global Health, Sydney, Australia; 2 Sydney Medical School, University of Sydney, Sydney, Australia; 3 Westmead Hospital, Sydney, Australia; 4 The University of Central Lancashire, Preston, United Kingdom; 5 Westmead Institute for Medical Research, Sydney, Australia; University of Manchester, UNITED KINGDOM

## Abstract

**Introduction:**

Many studies have now demonstrated the efficacy of text messaging in positively changing behaviours. We aimed to identify features and factors that explain the effectiveness of a successful text messaging program in terms of user engagement, perceived usefulness, behavior change and program delivery preferences.

**Methods:**

Mixed methods qualitative design combining four data sources; (i) analytic data extracted directly from the software system, (ii) participant survey, (iii) focus groups to identify barriers and enablers to implementation and mechanisms of effect and (iv) recruitment screening logs and text message responses to examine engagement. This evaluation was conducted within the TEXT ME trial—a parallel design, single-blind randomized controlled trial (RCT) of 710 patients with coronary heart disease (CHD). Qualitative data were interpreted using inductive thematic analysis.

**Results:**

307/352 (87% response rate) of recruited patients with CHD completed the program evaluation survey at six months and 25 participated in a focus group. Factors increasing engagement included (i) ability to save and share messages, (ii) having the support of providers and family, (iii) a feeling of support through participation in the program, (iv) the program being initiated close to the time of a cardiovascular event, (v) personalization of the messages, (vi) opportunity for initial face-to-face contact with a provider and (vii) that program and content was perceived to be from a credible source. Clear themes relating to program delivery were that diet and physical activity messages were most valued, four messages per week was ideal and most participants felt program duration should be provided for at least for six months or longer.

**Conclusions:**

This study provides context and insight into the factors influencing consumer engagement with a text message program aimed at improving health-related behavior. The study suggests program components that may enhance potential success but will require integration at the development stage to optimize up-scaling.

**Trial Registration:**

Australia and New Zealand Clinical Trials Registry, ACTRN12611000161921.

## Introduction

Coronary heart disease (CHD) remains the leading cause of death and disability worldwide.[1] Although the incidence of CHD has declined in regions such as the United States and United Kingdom due to population health improvements in risk factors, such as smoking cessation, healthier diet and increased physical activity,[2,3] the number of people surviving major cardiovascular events is increasing.[4] As such, a large percentage of hospitalisations for CHD are due to recurrent events and effective risk factor reduction, termed secondary prevention, can reduce these and therefore reduce the CHD burden.[5,6] Cardiac rehabilitation (CR) aims to deliver structured secondary prevention to people with CHD,[7] however, these services are consistently underused[8] and overall adherence to secondary prevention strategies remains suboptimal.[8,9,10] Consequently, new strategies in secondary prevention are required if we are to increase access and support for patients after hospital discharge.

In recent years, mobile phone text-message based interventions have arisen as a potential means of modifying health behaviors.[11] Recent studies have demonstrated the effectiveness of mobile phone text messaging to change individual health behaviours such as smoking cessation,[12,13,14] weight loss,[15] physical activity,[16] blood pressure lowering[17] and improved management of diabetes.[18] However, translation of these programs to public health or health service delivery has been limited and evidence suggests that reducing multiple risk factors concurrently is more effective in reducing cardiovascular events than targeting single risk factors.[19] In addition, very few studies included objective outcome measures and only the minority reported program evaluations with links to behavioural and psychological theory.

As recognised within the UK Medical Research Council framework for the development and evaluation of complex interventions, assessment of fidelity and causal mechanisms optimises the likelihood of implementation in clinical practice.[20] A recent ‘systematic mapping review’ that detailed the value of qualitative research alongside randomized controlled trials (RCTs)[21] found that it improved external validity, facilitated interpretation and provided an understanding of contextual factors that are important in terms of transferability and implementation.[21] Overall, there are numerous published studies reporting outcomes of text message based interventions but very few have have addressed and assessed their fidelity and how certain components can lead to behavioural change with psychological underpinning. Therefore, the aim of this study was to conduct a program evaluation (using mixed methods) to examine features and factors that contribute to the effectiveness of a successful text messaging program in terms of increased user/participant engagement, perceived usefulness (in relation to message frequency, content and personalization), behavior change and program delivery preferences so as to inform future research and implementation.

## Materials and Methods

### Design

We undertook a mixed methods approach within the Tobacco, EXercise and dieT messages (TEXT ME) single-blind RCT that aimed to reduce cardiovascular risk in 710 patients with CHD.[22,23] Results reporting clinical effectiveness of the TEXT ME intervention are reported elsewhere.[23] The intervention group received four text messages per week for six months (further detail on the messaging program below) in additional to usual care and the control group received usual care without the text message program. In summary, at six months, the intervention group had significantly lower LDL cholesterol, systolic blood pressure (BP), body mass index (BMI), smoking rates and significantly higher physical activity levels, compared to the control group.[23] Patients provided written informed consent and ethical approval was obtained from the Western Sydney Local Health District Human Research Ethics Committee.

The evaluation plan is summarised in Table 1. This program evaluation was designed alongside the TEXT ME trial and therefore data were collected in parallel to the RCT. The chosen methods follow a realist evaluation model which seeks to understand human choices and actions, within a systems context.[24] Qualitative and quantitative data sources comprised; (i) analytic data extracted directly from the text message software system, (ii) survey of participants to identify usefulness and engagement, (iii) focus groups to identify features influencing user engagement as well as barriers and enablers to program implementation in order to understand context and mechanisms of change and; (iv) recruitment screening logs to examine generalizability and engagement. In the context of this research, the term ‘engagement’ refers to the concept of user interaction with the program where it is determined by a combination of user interaction with the program and their viewpoints regarding what program features made them more likely to read and follow the advice. The combination of survey (all intervention participants invited) and focus groups allowed for synthesis of specific participant viewpoints in addition to exploration of more detailed responses via focus groups. The evaluation examined fidelity of the intervention, usefulness and user engagement as well as delivery model and content of the intervention.

**Table 1:** TEXT ME Program evaluation plan.

Objective	Data source/s
1. Assess the degree to which the TEXTME program was delivered as planned	Analytic data extracted from software, user surveys
2. Assess the potential reach of the program (within the context of a clinical trial)	Screening logs
3. Examine factors that influenced user engagement with the program	Focus groups and user surveys, analytic data, text message responses
4. Assess the program delivery in terms of timing, duration, frequency and content	Focus groups, user surveys
5. Links with psychological theory and behavior change techniques	Focus groups

We also examined the data for evidence of behavior change techniques and links with psychological theory based on a published taxonomy.[25] As reported previously, development of the text message program was based on a range of behavior change techniques including: the provision of information and encouragement; prompting about consequences, intention formation, monitoring self-behavior, and barrier identification; advice about setting graded tasks; and strategies aimed at relapse prevention and the use of prompting and cues.[26] Within this present evaluation, we explored the focus group data, using thematic analysis, for links to these theoretical frameworks so as to explore mechanisms and context associated with behavior change.

### Text messaging program

The text messaging program and its development process are detailed elsewhere.[22,23,27] The message program development[27] was based on behavior change techniques linked to the theoretical framework reported by Abraham and Michie.[25] In brief, it involved regular semi-personalised text messages providing advice, motivation, and information that aimed to improve general heart health, diet, physical activity and encourage smoking cessation (where relevant). Example messages are provided in Box 1. All participants received four messages per week for 24 weeks (smokers received fewer general heart health messages to accommodate smoking cessation messages). Messages were sent on four of five randomly selected week-days and arrived at random times during working hours. The messages were semi-personalized using baseline data about the participant’s preferred name and if the participant was vegetarian or a smoker. Participants were asked not to respond but they were informed the program would be managed by a computerized messaging engine and that they could reply with a message stating “STOP” if they wished to opt out of the program or they could contact the research coordinator listed on their patient information sheet.

BOX 1: Examples of text messages used in the TEXT ME trial[23]*

**Smoking**

[NAME], try identifying the triggers that make you want a cigarette & plan to avoid them

[NAME], for many it may take several attempts to quit, so keep trying

**Diet**

Did you know 90% of people don’t eat the recommended daily intake of vegetables (5 serves a day)?

Try avoiding adding salt to your foods by using other spices or herbs

**Physical Activity**

Hi [NAME], don’t forget physical activity is good for you! It reduces your risk of diabetes, heart attack, stroke, and their complications

Walking is cheap. It can be done almost anywhere. All you need is comfortable shoes & clothing

**General Cardiovascular Information**

Have you got a chest pain Action Plan [NAME]? Find ideas at http://www.heartfoundation.org.au/Pages/default.aspx

Studies show that stress, worry & loneliness can increase the risk of heart disease. Please talk to a health professional if you need help

*Reproduced with permission from JAMA 2015.*314(12)*:*1*. *Copyright (2015) American Medical Association*. *All rights reserved*.[23]

### Data sources

#### 1. Text-message software system analytics

We pre-specified in developing the TEXT ME message management system to record data on (TEXT ME Engine V1.0, Cardiology Department, Westmead Hospital) the total number of messages sent to each intervention participant and the time point that messages stopped being sent. In addition, any text message responses received from a participant throughout the course of the study were automatically logged and subsequently coded to enable identification of positive and negative responses as well as analysis of their content.

#### 2. User surveys

To examine acceptability and feasibility of the TEXT ME program, participants allocated to the intervention group were administered a survey (in confidence) after the blinded six month follow-up assessment. The survey comprised a total of 18 items: 15 required either 5-point Likert or yes/no categorical responses that explored utility, influence on behavior, potential for sharing messages and items addressing message frequency, timing and program duration. There were three items that provided an opportunity for free text feedback about usefulness and suggestions for improvement.

#### 3. Focus groups

At final follow-up, participants allocated to the TEXT ME intervention program were invited to participate in a focus group discussion. Stratified purposeful sampling was used to identify potential participants to ensure a variety of views were explored. This included ensuring participants in each focus group had a balance of gender, ethnicity, revascularization and history of cardiac rehabilitation participation. Recruitment for focus groups was consecutive until thematic saturation was achieved (determined when no new descriptive concepts were emerging). Standard focus group methods were used including the use of a facilitator (JR), scribe, setting of ground-rules and audio-recording.[28] A set of 12 open-ended prompts were used to guide and facilitate discussion around key topics (message delivery, content, usefulness and impact). Focus groups took place at the local hospital where participants were initially admitted. An additional independent person attended the focus groups to serve as a coordinator, manage recordings and take brief notes. The facilitator has more than 10 years of experience with CVD research, had no established relationship with participants and was not the Principal Investigator of the RCT.

#### 4. Screening logs

As part of the program, a screening log was kept to determine participation and reach. During the screening process to determine eligibility, basic details about potential participants and reasons for non-participation were recorded. This log was maintained by the study research assistant and were stored in a secure electronic database.

### Analysis

Demographic information and categorical results were summarised as means and proportions. Surveys were analysed for themes and the results were used to guide the development of focus group discussion points. To develop a coding framework based on the focus group data, a constant comparative method was used.[29,30] Two researchers (JR, GC) independently coded transcripts, line by line, and through two iterative stages a coding framework was developed. The coders were experienced clinician (nurse and allied health) researchers with expertise in CVD management, behaviour change and mixed methods research. Data checking was achieved by discussion between members of the multidisciplinary research team who are experts in the development and evaluation of secondary prevention programs. Where disagreements existed over the interpretation of data, these were resolved by consensus. Themes were then discussed amongst the full research group for review and further analysis. Throughout the coding process we constantly compared the content with previously coded data. This iterative process including collapsing and expanding various codes and themes until no new concepts were emerging. Direct quotations from the focus groups were used to illustrate some key emergent themes. In accordance with focus group methods, analysis examined the perspective of individuals and as well as emerging consensus between the participants around the issues being discussed.[29]

## Results

Of the 352 participants who received the text messaging program, 307 (87%) completed the user survey at six months. Their mean age was 58 (±8.9) years and most were male 255/307 (83%). A total of 25 participants took part in four focus groups and their mean age was 62 (±6) years and 15/25 (60%) were male. Focus groups were held at Westmead Hospital in Sydney between April 2013 and April 2014 for 1.5 hours duration each. Demographic and summary information about the survey and focus group participants is presented in Table 2 (see also S1 and S2 Tables).

**Table 2:** User survey (n = 307) and focus group (n = 25) participants’ demographic and medical history.

	User survey (n = 307)	Focus Groups (n = 25)
Age, mean (SD)	58 (9)	62 (6)
Male	255 (83%)	15 (60%)
Ethnic origin		
Australia/Europe	196/307 (64%)	15/25 (60%)
Asia	73/307 (24%)	5/25 (20%)
Arabic	28/307 (9%)	0/25 (0%)
Other	11/307 (4%)	5/25 (20%)
Years of education		
< 10 years	87/307 (28%)	2/25 (8%)
10–12 years	133/307 (43%)	10/25 (40%)
≥ 13 years	86/307 (28%)	13/25 (52%)
Previous myocardial infarction	101/307 (33%)	16/25 (64%)
Prior revascularization		
CABG	48/307 (16%)	2/25 (8%)
PCI	221/307 (72%)	14/25 (56%)
Both CABG and PCI	15/307 (5%)	2/25 (8%)
Attended cardiac rehabilitation	140/307 (46%)	8/25 (64%)

SD, standard deviation; CABG, coronary artery bypass graft; PCI, percutaneous coronary intervention

In terms of reach, a total of 1301 patients with CHD were approached to participate in the TEXT ME trial (of which 710 were enrolled into the trial and 352 were randomised to the intervention group). Of those approached, the majority owned a mobile phone (1036/1301, 80%) and very few had privacy concerns (8/1301, 0.6%) or were unable to communicate via text message (9/1301, 0.7%). Of the 591 who did not participate reasons included; not owning a mobile phone (44.8%, 265/591), not able to read messages in English language (34.7%, 205/591), decline to participate in research (11.8%, 70/591) and other non-specific reasons (8.6%, 51/591) [23]. In terms of fidelity, based on analytic data extract from the software system, 96% (338/352) of participants allocated to the intervention group received all 98 scheduled messages during the six months. During the intervention period, participants were asked not to respond to messages they received, however, we still received a total of 308 text message replies from 116 unique mobile telephone numbers. Most of the replies received were related to saying thank you (39%) but others were to provide feedback about behavior (18%), to make a general comment (16%), to unsubscribe (12%), request further information (7%), extraneous comment unrelated to the program (6%) or were complaints (2%).

### Factors influencing user engagement with the text message program

**Fig 1:**
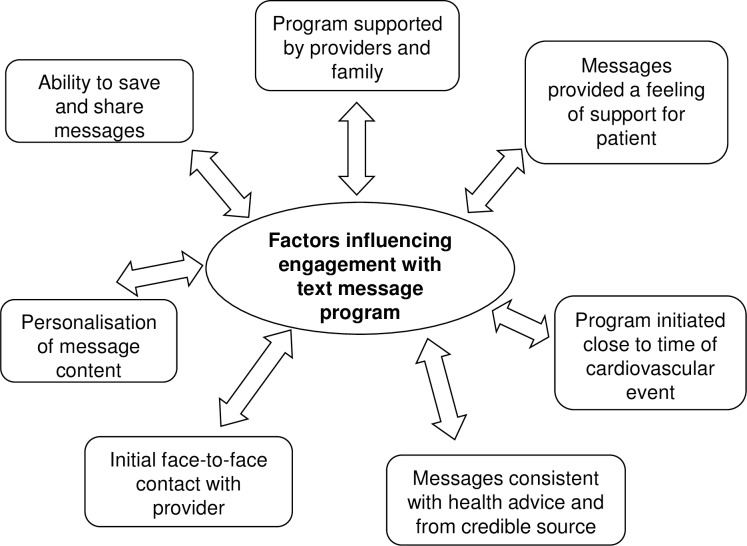
Overview of factors influencing engagement with the text message program.

Analysis of qualitative data collected in the focus groups confirmed a high level of user engagement. Seven themes emerged as factors influencing program user engagement (Fig 1). These included; (i) the ability to save and share messages, (ii) having the support of providers and family, (iii) a feeling of support through receipt of the program, (iv) the program being initiated close to the time of a cardiovascular event, (v) personalization of the messages, (vi) opportunity for initial face-to-face contact with a provider and (vii) that the message content was consistent with previous advice and from a credible source. Table 3 provides direct quotations from focus group participants illustrating the identified themes and factors influencing engagement. User engagement was also demonstrated by the fact that despite participants being asked not to respond to messages we received a total of 403 text message replies from 134 distinct mobile telephone numbers during the six month intervention period. Most of the replies received were related to saying thank you (30%), to provide feedback about a healthy behavior (14%) or to make a general comment (12%). As further evidence of engagement, in the user survey, 96% of participants reported that during the course of the program they read at least three-quarters of all text messages. In addition, 54% of survey participants and 55% of focus group participants reported that they saved or shared the text messages and 21% left the messages in their inbox.

**Table 3:** Quotations illustrating factors influencing user engagement with text message program.

**1. Potential to save and share messages**
*“I read them and kept them” (“P”*, *male*, *61*, *FG4”)*
*“It was also good for educating the family” (“G”*, *male*, *58*, *FG2)*
*“I forwarded the messages to a few friends [overseas]” (“T”*, *female*, *49*, *FG1)*
*“I kept everything in a thread*, *so if I want to go back and read it later*, *I can find it” (“D”*, *male*, *65*, *FG2)*
*“I got a message come through … with a recipe to prepare a couple bits of fresh fish and veggies*. *I prepared the dish and took photograph of it and put it on Facebook” (“D”*, *male*, *65*, *FG2)*
*“I haven’t delete mine until I have written them all down” (“G”*, *female*, *63*, *FG3)*
**2. Provider, clinician and family support**
*“I spoke to the cardiologist about it*. *He thought it was a good idea and was very supportive” (“S”*, *male*, *67*, *FG2)*
*“My children loved it and thought it was great*, *the way of receiving health information via texts*. *‘Welcome to the 21*^*st*^ *century’” (“B”*, *female*, *60*, *FG1)*
*“I told my GP and cardiologist and my nutritionist*. *She was happy about it*. *She found that the messages great*, *they were simple but to the point” (“C”*, *female*, *61*, *FG2)*
*“When my doctor recommended*, *I said yes” (“M”*, *female*, *53*, *FG3)*
**3. Feeling of support**
*“The on-going contact encouraged me to stay on the straight way I suppose (“B”*, *female*, *60*, *FG1)”*
*“It is like a link back to hospital…*.*it gives a good feeling just knowing that there is someone else that cares and kept an eye on it/you*, *felt like a good link” (“K”*, *male*, *53*, *FG1)*
*“I don’t have my own GP; I see whoever I want every time*, *having some contact is what is good about it” (“B”*, *female*, *60*, *FG1)*
*“This calmed me down a lot and gave me reassurance” (“J”*, *male*, *64*, *FG1)*
*“It was nice to know that somebody cared” (“W”*, *male*, *68*, *FG1)*
**4. Program being initiated close to the time of a cardiovascular event**
*“You have to have had an event to feel the motivation” (“T”*, *female*, *49*, *FG1)*
*“I think you are more open to accept any new information [after a heart attack]” (“M”*, *female*, *73*, *FG3)*
*“After the event*, *you are looking for any sort of information that can prevent that sort of thing from happening again” (“J”*, *male*, *68*, *FG3)*
*“In the first few weeks*, *you still have the emotional side of having a heart attack*. *So I think you need to have a couple of weeks to settle down before you get the messages” (“J”*, *male*, *70*, *FG4)*
*“I think you need to get them when you are still scared*. *I think you are probably scared for a month to 6 weeks” (“P”*, *male*, *61*, *FG4)*
**5. Personalization of the messages**
*“Every so often the messages had something to do with me…I was very interested in that part” (“J”*, *male*, *64*, *FG1))*
*“I found them very personal*. *It was good that they came with your nickname*, *which was the same as my friends always use” (“B”*, *female*, *60*, *FG1)*
*“The first letters in that text message*, *saying hi Jim*, *you knew it wasn’t from the local Thai place” (“S”*, *male*, *67*, *FG2)*
**6. Opportunity for initial face-to-face contact with provider**
*“Without seeing a person*, *you don’t imagine what you need and what you want—when you see her and talk to her*, *it gives yourself confidence” (“T”*, *male*, *62*, *FG4)*
*“It’s really good*, *because she talks to you individually*. *It feels that someone is looking after you*. *… So all the time you are looking forward to the message” (G”*, *male*, *64*, *FG4)*
*“And the other thing is*, *if I didn’t speak to (research assistance and clinician)*, *if she didn’t come and see me*, *and I get the text messages*, *maybe I wouldn’t pay that much attention either” (“T”*, *male*, *62*, *FG4)*
**7. Message content was consistent with previous advice and from credible source**
*“I imagined it coming from the hospital*, *because we had prior knowledge of it coming from the hospital” (“D”*, *male*, *65*, *FG2)*
*“It was hand in hand with the dietician [information]” (“C”*, *female*, *51*, *FG2)*
*“It’s trustworthy information coming from [hospital]” (“F”*, *male*, *66*, *FG3)*
*“I thought the information was pretty consistent with everything else I have been told*, *which is good (“T”*, *female*, *49*, *FG1)*

### Program delivery and perceived usefulness

Overall, the text message-based program was considered to be convenient, instant and private. Focus group and survey participants were consistently positive about the program and the potential for continuation with illustrative quotations as follows.

“Think it is a good choice to offer to everyone in every hospital” (“C”, male, 65, FG1)

“This should continue, this program is excellent” (“C”, female, 51, FG1)

“I think that the program has a great positive impact….it should be available to everyone” (“D”, male, 61, FG3)

These findings are supported by previously reported survey data demonstrating that most participants reported the program was useful (91%), easy to understand (97%) and motivating (77%).[23] Also, when asked about behavior related to specific risk factors as a result of the messages, 81% reported adopting a more healthy diet, 73% reported they increased their physical activity and 76% reported that the messages reminded them to take their prescribed medications.[23] These self-reported improvements directly align with significant quantitative improvements in cardiovascular risk factors including biomedical measures of BP and cholesterol as well as validated questionnaires assessing physical activity and nutrition.[23]

Using qualitative analysis, we specifically evaluated the program barriers and enablers in relation to characteristics of (i) message content, (ii) delivery frequency, (iii) message delivery timing, (iv) program duration and (v) one-way communication.

#### i. Message content

In focus groups, the consensus was that the positive content, practical nature and variety of message content was good, easy to understand and included nothing offensive. In terms of content, there was a clear theme that the diet and physical activity messages were considered the most valuable (Table 4). However, participants found the medication and clinical information useful as well (Table 4). For improvement, participants suggested more content related to eating out and mental health and that the program could allow for variation to pre-defined data, for example, if they stopped smoking during the course of the program. In total, 96% of survey participants reported that the language of the text messages was appropriate with the minority reporting that the messages were too casual (2%) or too formal (2%).

**Table 4:** Quotations illustrating participant views relating to message content and perceived usefulness.

**Intention to change lifestyle (usefulness of physical activity, diet and clinical messages)**
*“The food and exercise messages were great because both of them are really important to the person who had a heart problem” (“T”*, *male*, *62*, *FG4)*
*“It is amazing how my eating has changed” (“G”*, *male*, *58*, *FG2)*
*“It did encourage me to join up in a gym” (“F”*, *male*, *66*, *FG3)*
*“The dietary is number one and then the ones about blood pressure” (“F”*, *male*, *66*, *FG3)*
*“It is good to receive some information about what the medications do*, *because sometimes I received information that the GP didn’t tell me about*, *which was really good” (“T”*, *female*, *49*, *FG1)*
**Importance of practical information and variety**
*“I liked the practical messages*, *for example about orange juice and how much sugar is in it” (“A”*, *male*, *50*, *FG4)*
*“The liked the dietary ones with the practical examples*, *like eating more nuts” (“M”*, *female*, *73*, *FG3)*
*“The variety of the messages was good*, *they were all different” (“S”*, *male*, *67*, *FG2)*
*“Good range of information on lots of different topics” (survey participant*, *male*, *49)*
**Suggestions for content improvement**
*“Maybe more information about mental health and stress” (“T”*, *female*, *49*, *FG1)*
*“It would be a good idea to send out a list of recommended restaurants that served the correct meals…*. *for different kind of restaurants like Indian*, *Thai etc” (“S”*, *male*, *67*, *FG2)*
*“Smoking messages irritated me cause I have given up smoking” (survey participant*, *male*, *38)*
*“I'm low English so some messages were tricky” (survey participant*, *male*, *53)*
*“Lots of information was similar*, *not a bad thing though” (survey participant*, *male*, *51)*

#### ii. Message delivery frequency

In the user survey, 87% of participants reported that the number of messages they received (4 per week) was appropriate with 8% reporting that there were too few and 5% reporting there were too many. When we explored this further in the focus groups, most participants felt the random timing of messages was important and that the frequency was generally considered ideal. Participants suggested that too many messages per day would be overwhelming and could lead to reduced engagement. Some of the comments related to message frequency include the following;

“Even 7 days a week would be fine and wouldn’t bother me” (“T”, female, 49, FG1)

“I think even 5 are alright” (“F”, 59, male, FG1)

#### iii. Message timing

Most survey and focus group participants reported that the timing of message receipt was appropriate with illustrative quotes as follows.

“It is good that the messages weren’t sent in the weekends” (“C”, female, 51, FG2)

‘I think receiving the text message late at night, like 10pm, is not what you want” (“J”, male, 62, FG3)

“I think Monday to Friday is perfect” (“K”, 60, female, FG1)

On further analysis in the focus groups, participants clearly supported the random delivery timing and felt this helped keep them interested and engaged.

“I thought it was good that it came random, which makes it not programed into you” (“F”, male, 59, FG1)

“It was good that the messages arrived at different times of day unexpectedly” (“B”, female, 60, FG1)

#### iv. Program duration

The primary theme emanating from the focus groups in relation to program duration was that it could have been longer than six months but with a lower message frequency after month six with illustrative quotations such as;

“Once it finished, I found myself failing again” (“D”, male, 61, FG3)

“One day they stopped, that was that last one. I would say I missed them in some way, because I was used to getting them” (“J”, male, 68, FG3)

“Maybe you can drop down to 2 messages a week for the second 6 months” (“S”, male, 67, FG2)

“Would it be possible to run the program for more than 6 months” (“S”, male, 67, FG2)

These above findings were consistent with the findings of the user survey where 79% felt the six month duration was appropriate, 16% felt it was too short and 5% felt it was too long. Considering the survey and focus group data together, there was a theme that program length of at least 6 months was ideal with a possible option for a longer duration.

#### v. One way communication

There were mixed views about the one-way communication. Overall, there was consensus that it would be ideal to offer a two-way communication feature for those who are interested but two-way communication would not be required for most participants.

“I’m not a communicator, so I like the idea of being passive. I find it is fine like it is” (“D”, male, 61, FG3)

“I think it would be absolutely brilliant to be able to communicate with someone” (“D”, male, 65, FG2)

“Perhaps in a future program, like communicating back once in every 6 months or once in 3 months” (“A”, male, 50, FG4)

“Maybe every 9 or 6 months to have a phone call, maybe some volunteers who have had heart attacks and that you can talk to them if you have some questions” (“J”, male, 70, FG4)

### Links with psychological theory and behavior change techniques

Three dominant theoretical frameworks were identified as potentially contributing to the observed behavior change and the TEXT ME program. The Information-Motivation-Behavior Skills Theoretical Model[31] could primarily explain the observed behavior change associated with the TEXT ME program. This theory states that if a person is well-informed, motivated to act, and they have the skills and confidence to take action, they are more likely to initiate and maintain health-promoting behaviors that produce positive outcomes.[31] In the context of our messaging program, a theme emerged that participants felt motivated and engaged by the program when it provided credible and meaningful information with illustrative quotations links with this theory including:

“So the messages prompt me to research all my medications” (“D”, male, 65, FG2)

“I actually researched how many calories you should eat in a day and what’s in everything and I set a spreadsheet and I started calculating what you should eat and what you shouldn’t eat.” (“A”, male, 50, FG4)

“Any info is good. After just having a heart attack I wanted more info about what I should be doing and what shouldn’t” (“T”, female, 49, FG1)

“It is good to receive some information about what the medications do, because sometimes I received information that the GP didn’t tell me about, which was really good” (“T”, female, 49, FG1)

“The messages gave me some insight into new things that I had never thought of, trying different foods” (“G”, male, 58, FG2)

Although the program was a one-way text message communication system, qualitative data suggest that Social-Cognitive Theory explains some of the observed behavior change. Social cognitive theory[32] suggests that human behavior is determined by a combination of cognitive, personal and environmental influences. These reflect an interaction between thought, affect and action where expectations, beliefs, self- perceptions, goals and intentions give shape and direction to behavior.[33] Further, behaviour is modified based on whether the individual has high or low self-efficacy toward the behavior, the response a person receives after performing the behavior and aspects of the environment that improve self-efficacy including the setting of achievable goals. In the current text messaging program, social recognition, improved self-efficacy, the provision of achievable task-setting suggestions, practical advice and positive reinforcement were all built into the program[27] and quotes illustrating their successful implementation are below.

“I found the program encouraging” (“B”, female, 60, FG1)

“It gives a good feeling just knowing that there is someone else who cares and kept an eye on it/you like a good link” (“K”, male, 58, FG1)

“It made me feel good when sometimes I already did my exercise and then got a message about it. And then I was like ‘Yes, beat you’” (“B”, female, 60, FG1)

Operant and its predecessor Classical Conditioning are theoretical frameworks[34,35] for explaining behavior and this concept of ‘respondent condition’ appeared to emerge from the focus groups where participants responded to the repeated messaging within the program as a whole rather than the immediate content of individual messages. This type of conditioning was first reported by Ivan Pavlov where he demonstrated that dogs learned a stimulus-response connection in response to repeated stimuli such that a previously neutral stimulus eventually elicits a response.[35] Our qualitative data suggest that over time participants became ‘conditioned’ towards healthy behaviors irrespective of the specific content of the individual messages received at a certain time. That is, through repeated presentation of a stimulus (in this case cardiovascular health related text messages) the participants eventually learned to associate the stimulus with overall cardiovascular health behaviour and consequently messages about diet also resulting in improved physical activity and vice versa. The notion that the program evoked a feeling of feeling “guilty” could also contribute to conditioning where the feeling of guilt is a noxious stimuli associated with ‘unhealthy’ behaviour. Illustrative quotes highlighting this concept include;

“It wasn’t particularly about what the message said that day” (“C”, female, 51, FG2)

“They always popped up before I was about to eat something naughty” (“W”, male, 68, FG1)

“I don’t actually go for context of the message, but look for the little nudge. The little things that nudge you to do the right things” (“D”, male, 65, FG 2)

“When you see the text message you might be a little bit more conscious about what you are eating” (“T”, male, 62, FG4)

“If I didn’t walk for the past 3 or 4 nights, because it was too hot or raining, you made a bit more effort to do it, you felt a bit guilty. And the same with checking your cholesterol and about what you are eating, think about the portions etc. It is an on-going thing, a general thing. But the reminder or the nag that was the key” (“S”, male, 76, FG2)

“I love chocolate and one time I was in the aisle of [shop name] thinking about getting a snack… then suddenly I received a message of TEXT ME saying: Hi [name], feeling like a snack? A banana or a piece of fruit! It really gave a big brother feeling to me, how could they know I was thinking about picking a snack? I thought well forget it lets go and walked away with a smile on my face. I got busted” (“B”, female, 60, FG1)

## Discussion

This process evaluation found that patients with CHD were engaged with and positive about a text message program aimed at supporting behaviour change. Results suggest that engagement was increased by several factors including the program being initiated close to the time of hospitalisation, the consistency of the messages with prior in-hospital education and management, having the opportunity for face-to-face contact at program commencement and the personalization of messages. We found features that impacted on behavioural change were the positive content, the sense of feeling supported, the delivery time being random and that most participants felt program duration could have been longer than six months. Through thematic analysis this research has identified several potential drivers of behavior change within a text messaging program and therefore facilitates understanding of components of the intervention that may increase effectiveness.

The primary goal of this evaluation was to help with understanding why text message-based programs for people with CHD are effective, the ingredients for success and how engagement can be optimized so as to inform development of other text message programs aimed at behavioural change in patients with other type of chronic disease. Overall, an important role for program evaluations is to examine the quantity and quality of what was actually implemented in practice and why.[20,21] Through understanding of barriers and enablers as well as reach and fidelity, program evaluations can inform future implementation of similar interventions and facilitate interpretation of intervention outcomes.[21] It is well known that exploring issues such as acceptability and perceived usefulness from a consumer perspective enables program developers and those responsible for implementation to better understand intervention delivery and reach.[20] The use of a mixed methods approach allows the integration of numerical data with perspective gained from a qualitative approach.[36] In common with our results, other mixed methods studies of health-behavior interventions have found that most patients want disease specific information and self-management strategies.[37] Our intervention was based on behavioural theory and was developed via a formal process involving consumers. The presented qualitative data provide a deeper understanding of the mechanisms associated with the observed program effectiveness.[23] Such findings will be available for development of future programs targeting patients with CVD and other health conditions so as to inform policy and upscaling of similar programs designed to improve behaviour change.

Very few studies have reported qualitative evaluations alongside a RCT of a text message-based intervention for improving health-related behavior. One study tested the effectiveness of prompts sent via text message in terms of improving BP management via adherence to clinic visits and treatment.[38] The qualitative evaluation was conducted via focus groups aiming to explore trial participants’ experiences and identified barriers to intervention delivery. The study concluded that the message program was acceptable, relevant, provided practical support for patients and that intervention success coincided with participant readiness for change.[38] Similar to our study, Leon et al[38] reported that most participants found the text messages with practical tips for healthy living were most useful, that the messages improved adherence, that participants were comfortable receiving the messages and that a polite and respectful tone was important.

Understanding the theory associated with behavior change allows consideration of hypothesized causal processes involved.[39] In the case of the TEXT ME messaging program, the messages themselves were based on theory[27] and understanding the factors influencing engagement and the mechanisms associated with the observed clinical outcomes[23] is important for scalability, uptake and future implementation. Gaining an understanding of these mechanisms informs development of future content, delivery frequency and structure. Insight into the mechanisms of behavior change augments the clinical outcomes and ultimately enables a deeper understanding of the effectiveness. This level of analysis is important because ultimately a person’s behaviour is the result of their individual or collective action and it is a key determinant of health.[40] This concept is particularly important in relation to CHD where lifestyle risk factors are leading contributors to morbidity and mortality.[24] The behaviours that cause and contribute to CHD are common and changing them often means a person is required to make multiple and long-term changes to their everyday behavior. For example, a person may be recommended to stop smoking, increase their physical activity level and take daily medications for the remainder of their life. A National Institute for Health and Clinical Excellence guideline highlights the importance of identification and evaluation of mechanisms associated with behaviour change and that mixed method ethnographic research, longitudinal studies and qualitative approaches are needed.[41] Gaining an understanding of underlying theoretical factors influencing behaviour help establish why an intervention is working so as to inform decisions relating to implementation and improvement [41].

While this mixed methods evaluation provides significant insight into the potential mechanisms of intervention effectiveness and opportunities for optimising engagement, the study is not without limitations. Firstly, the sample was recruited from within a RCT from a tertiary hospital which might limit the generalisability of the intervention, however this hospital has broad socioeconomic and multicultural representation. Secondly, our sample size for women was small and therefore examination of gender differences requires further investigation. It is acknowledged that we did not stratify for age and that there were more people with higher years of education participating in the focus groups and that our findings will vary between individuals and naturally certain groups should not be denied access to potential interventions. The qualitative nature of this research enables features to be examined in detail and allows consideration of human experience. Future research is needed to identify the effectiveness of various program design features. For example, to specifically determine whether it is ideal to offer one-way or two-way communication or whether this should be offered to participants by choice. In addition, future research could clearly define the ideal message delivery frequency, timing and the ideal program duration.

## Conclusions

This process evaluation found that patients with CHD were engaged with and positive about a text message program aimed at supporting behaviour change. Exploring the theoretical frameworks provides insight into the causal pathway between the text-message intervention, behavior change and impact on risk factor measures. Gaining an understanding of the factors that influence engagement with the program as well as the advantages and disadvantages of various program characteristics can inform the development of other programs, identifies the key elements important to maintain in larger-scale programs and gives clues to the directions of future research to improve programs and support their sustained effectiveness.

## Supporting Information

S1 Table Focus Group Demographic information. (DOCX)

S2 Table Survey Group Demographic information (DOCX)
