# Supplementary material for: Factors Influencing Engagement, Perceived Usefulness and Behavioral Mechanisms Associated with a Text Message Support Program
Source: PLoS One. 2016 Oct 14;11(10):e0163929. doi: 10.1371/journal.pone.0163929 (PMC5065147; doi:10.1371/journal.pone.0163929)
Supplement: S1 Table — (DOCX) [file pone.0163929.s001.docx]

| S1 Table: Focus Group Demographic information | | | | | | | |
| --- | --- | --- | --- | --- | --- | --- | --- |
| Focus group ID | Age | Gender | Country of birth | Education level | Prior MI | Prior revascularization | Cardiac rehabilitation |
| 1 | 65 | 1 | SRI LANKA | 2 | 1 | 2 | 0 |
| 2 | 58 | 0 | AUSTRALIA | 2 | 1 | 1 | 0 |
| 3 | 67 | 0 | UK | 2 | 0 | 1,2 | 0 |
| 4 | 51 | 1 | AUSTRALIA | 2 | 0 | 0 | 0 |
| 5 | 60 | 0 | AUSTRALIA | 3 | 0 | 2 | 0 |
| 6 | 59 | 0 | SRI LANKA | 1 | 0 | 0 | 0 |
| 7 | 49 | 1 | NEW ZEALAND | 2 | 1 | 0 | 0 |
| 8 | 60 | 1 | AUSTRALIA | 3 | 0 | 0 | 0 |
| 9 | 60 | 1 | AUSTRALIA | 2 | 0 | 2 | 0 |
| 10 | 66 | 0 | INDIA | 4 | 0 | 2 | 0 |
| 11 | 58 | 0 | AUSTRALIA | 2 | 1 | 0 | 0 |
| 12 | 64 | 0 | FRANCE | 3 | 1 | 2 | 1 |
| 13 | 68 | 0 | USA | 2 | 1 | 2 | 0 |
| 14 | 75 | 1 | MALTA | 1 | 1 | 0 | 0 |
| 15 | 66 | 1 | PHILIPPINES | 4 | 1 | 2 | 1 |
| 16 | 73 | 0 | AUSTRALIA | 5 | 0 | 2 | 1 |
| 17 | 50 | 1 | AUSTRALIA | 4 | 1 | 1 | 1 |
| 18 | 63 | 1 | ENGLAND | 3 | 1 | 2 | 0 |
| 19 | 62 | 1 | MALAYSIA | 3 | 1 | 2 | 1 |
| 20 | 70 | 1 | AUSTRALIA | 3 | 1 | 2 | 0 |
| 21 | 58 | 1 | Thailand | 2 | 1 | 0 | 0 |
| 22 | 61 | 1 | NEW ZEALAND | 5 | 1 | 1,2 | 1 |
| 23 | 61 | 1 | USA | 4 | 1 | 2 | - |
| 24 | 63 | 2 | AUSTRALIA | 2 | 0 | 2 | 1 |
| 25 | 68 | 1 | AUSTRALIA | 3 | 1 | 2 | 1 |
