# Supplementary material for: Factors Influencing Engagement, Perceived Usefulness and Behavioral Mechanisms Associated with a Text Message Support Program
Source: PLoS One. 2016 Oct 14;11(10):e0163929. doi: 10.1371/journal.pone.0163929 (PMC5065147; doi:10.1371/journal.pone.0163929)
Supplement: S2 Table — (DOCX) [file pone.0163929.s002.docx]

| S2 Table: Survey Group Demographic information | | | | | | | | |
| --- | --- | --- | --- | --- | --- | --- | --- | --- |
| AGE | GENDER | ETHNICITY | YEARS OF EDUCATION | PRIOR MI | PRIOR CABG | PRIOR PCI | PRIOR CABG + PCI | CARDIAC REHABILITATION |
| 66 | 0 | 1 | 16 | 0 | 0 | 0 | 0 | 0 |
| 68 | 0 | 10 | 7 | 0 | 1 | 0 | 1 | 1 |
| 42 | 1 | 10 | 10 | 1 | 0 | 1 | 1 | 0 |
| 57 | 1 | 10 | 13 | 0 | 0 | 0 | 0 | 0 |
| 75 | 0 | 10 | 10 | 1 | 0 | 0 | 0 | 0 |
| 52 | 0 | 10 | 10 | 1 | 0 | 0 | 0 | 0 |
| 66 | 0 | 10 | 10 | 0 | 1 | 0 | 1 | 0 |
| 42 | 0 | 5 | 12 | 0 | 0 | 0 | 0 | 0 |
| 53 | 0 | 8 | 11 | 0 | 0 | 1 | 1 | 1 |
| 54 | 0 | 7 | 10 | 1 | 0 | 0 | 0 | 0 |
| 67 | 0 | 10 | 10 | 0 | 0 | 0 | 0 | 0 |
| 53 | 1 | 10 | 11 | 0 | 0 | 0 | 0 | 0 |
| 67 | 0 | 10 | 10 | 0 | 0 | 0 | 0 | 0 |
| 59 | 0 | 10 | 16 | 0 | 0 | 1 | 1 | 1 |
| 58 | 0 | 10 | 14 | 0 | 0 | 0 | 0 | 0 |
| 59 | 0 | 10 | 11 | 0 | 0 | 0 | 0 | 1 |
| 76 | 0 | 10 | 10 | 0 | 1 | 0 | 1 | 0 |
| 54 | 0 | 7 | 11 | 0 | 1 | 0 | 1 | 1 |
| 46 | 1 | 10 | 11 | 0 | 0 | 0 | 0 | 0 |
| 57 | 0 | 10 | 11 | 1 | 0 | 1 | 1 | 0 |
| 58 | 0 | 10 | 16 | 1 | 1 | 0 | 1 | 1 |
| 46 | 0 | 10 | 11 | 0 | 0 | 0 | 0 | 0 |
| 51 | 1 | 10 | 11 | 0 | 0 | 0 | 0 | 1 |
| 57 | 0 | 1 | 9 | 0 | 0 | 0 | 0 | 1 |
| 59 | 0 | 10 | 9 | 1 | 0 | 0 | 0 | 0 |
| 45 | 1 | 10 | 11 | 0 | 0 | 0 | 0 | 0 |
| 60 | 0 | 10 | 11 | 1 | 0 | 1 | 1 | 0 |
| 66 | 0 | 1 | 13 | 1 | 0 | 1 | 1 | 0 |
| 60 | 1 | 10 | 16 | 0 | 0 | 0 | 0 | 0 |
| 63 | 0 | 5 | 8 | 0 | 0 | 1 | 1 | 0 |
| 60 | 1 | 10 | 11 | 0 | 0 | 1 | 1 | 1 |
| 67 | 0 | 10 | 15 | 1 | 0 | 0 | 0 | 0 |
| 61 | 0 | 2 | 16 | 0 | 0 | 1 | 1 | 1 |
| 63 | 0 | 1 | 11 | 0 | 0 | 1 | 1 | 0 |
| 71 | 0 | 10 | 20 | 0 | 0 | 0 | 0 | 0 |
| 57 | 0 | 10 | 8 | 1 | 0 | 0 | 0 | 0 |
| 57 | 0 | 10 | 11 | 0 | 1 | 0 | 1 | 0 |
| 62 | 1 | 10 | 13 | 1 | 0 | 1 | 1 | 0 |
| 55 | 1 | 10 | 17 | 1 | 0 | 1 | 1 | 0 |
| 63 | 0 | 10 | 11 | 0 | 0 | 0 | 0 | 1 |
| 70 | 0 | 10 | 12 | 0 | 0 | 0 | 0 | 0 |
| 57 | 0 | 10 | 11 | 0 | 0 | 0 | 0 | 1 |
| 60 | 1 | 10 | 13 | 0 | 0 | 1 | 1 | 0 |
| 76 | 0 | 5 | 17 | 1 | 0 | 1 | 1 | 1 |
| 50 | 0 | 10 | 12 | 0 | 0 | 1 | 1 | 0 |
| 63 | 0 | 10 | 16 | 0 | 0 | 1 | 1 | 1 |
| 45 | 0 | 10 | 16 | 0 | 0 | 1 | 1 | 0 |
| 69 | 1 | 10 | 9 | 0 | 1 | 0 | 1 | 0 |
| 61 | 1 | 19 | 10 | 0 | 0 | 0 | 0 | 1 |
| 54 | 0 | 7 | 11 | 0 | 0 | 1 | 1 | 1 |
| 54 | 0 | 10 | 10 | 0 | 0 | 0 | 0 | 0 |
| 41 | 0 | 10 | 13 | 0 | 0 | 1 | 1 | 0 |
| 54 | 0 | 7 | 18 | 0 | 0 | 0 | 0 | 0 |
| 63 | 0 | 10 | 10 | 0 | 1 | 0 | 1 | 0 |
| 60 | 1 | 10 | 9 | 0 | 0 | 0 | 0 | 1 |
| 52 | 0 | 7 | 11 | 0 | 0 | 1 | 1 | 1 |
| 43 | 0 | 10 | 13 | 1 | 0 | 1 | 1 | 0 |
| 62 | 0 | 5 | 18 | 0 | 1 | 1 | 2 | 1 |
| 69 | 1 | 10 | 13 | 0 | 0 | 1 | 1 | 0 |
| 51 | 0 | 5 | 17 | 0 | 0 | 1 | 1 | 0 |
| 65 | 0 | 1 | 20 | 1 | 0 | 1 | 1 | 1 |
| 62 | 0 | 10 | 11 | 1 | 1 | 0 | 1 | 0 |
| 62 | 0 | 7 | 9 | 1 | 0 | 1 | 1 | 1 |
| 57 | 0 | 10 | 10 | 0 | 0 | 0 | 0 | 0 |
| 57 | 1 | 10 | 10 | 0 | 0 | 1 | 1 | 0 |
| 51 | 0 | 1 | 11 | 1 | 0 | 1 | 1 | 0 |
| 41 | 0 | 10 | 12 | 0 | 1 | 0 | 1 | 0 |
| 58 | 1 | 10 | 8 | 0 | 0 | 1 | 1 | 0 |
| 48 | 0 | 10 | 15 | 1 | 0 | 0 | 0 | 1 |
| 55 | 1 | 5 | 10 | 0 | 1 | 0 | 1 | 0 |
| 58 | 1 | 10 | 13 | 0 | 0 | 0 | 0 | 0 |
| 47 | 1 | 10 | 11 | 0 | 0 | 1 | 1 | 1 |
| 56 | 0 | 19 | 12 | 0 | 1 | 0 | 1 | 1 |
| 49 | 1 | 10 | 13 | 0 | 0 | 1 | 1 | 0 |
| 52 | 0 | 10 | 11 | 1 | 1 | 0 | 1 | 1 |
| 43 | 0 | 7 | 12 | 1 | 0 | 0 | 0 | 0 |
| 54 | 0 | 10 | 16 | 0 | 0 | 1 | 1 | 1 |
| 49 | 0 | 1 | 16 | 1 | 0 | 1 | 1 | 1 |
| 63 | 0 | 9 | 16 | 0 | 0 | 0 | 0 | 1 |
| 61 | 0 | 10 | 17 | 0 | 0 | 1 | 1 | 0 |
| 60 | 0 | 10 | 12 | 1 | 0 | 1 | 1 | 0 |
| 45 | 0 | 5 | 16 | 0 | 0 | 0 | 0 | 0 |
| 49 | 1 | 1 | 11 | 0 | 0 | 1 | 1 | 1 |
| 54 | 0 | 7 | 18 | 0 | 0 | 1 | 1 | 1 |
| 71 | 0 | 10 | 10 | 0 | 0 | 1 | 1 | 0 |
| 45 | 0 | 10 | 13 | 0 | 0 | 1 | 1 | 1 |
| 58 | 0 | 5 | 14 | 0 | 0 | 1 | 1 | 0 |
| 63 | 0 | 10 | 8 | 1 | 1 | 0 | 1 | 1 |
| 61 | 0 | 10 | 9 | 0 | 0 | 1 | 1 | 1 |
| 51 | 0 | 10 | 10 | 0 | 0 | 1 | 1 | 0 |
| 43 | 0 | 1 | 24 | 0 | 0 | 1 | 1 | 0 |
| 64 | 0 | 10 | 12 | 0 | 0 | 0 | 0 | 0 |
| 64 | 0 | 10 | 11 | 1 | 0 | 1 | 1 | 1 |
| 67 | 0 | 10 | 7 | 0 | 0 | 1 | 1 | 1 |
| 48 | 0 | 5 | 10 | 0 | 0 | 0 | 0 | 1 |
| 46 | 0 | 1 | 16 | 0 | 0 | 0 | 0 | 1 |
| 52 | 0 | 5 | 8 | 1 | 0 | 1 | 1 | 1 |
| 58 | 0 | 1 | 12 | 0 | 0 | 1 | 1 | 1 |
| 43 | 0 | 1 | 17 | 0 | 0 | 1 | 1 | 0 |
| 48 | 0 | 10 | 12 | 0 | 0 | 1 | 1 | 1 |
| 50 | 0 | 10 | 12 | 1 | 0 | 1 | 1 | 0 |
| 44 | 1 | 5 | 17 | 0 | 0 | 0 | 0 | 0 |
| 69 | 0 | 10 | 9 | 0 | 0 | 1 | 1 | 0 |
| 70 | 0 | 10 | 11 | 0 | 0 | 0 | 0 | 1 |
| 58 | 1 | 10 | 17 | 0 | 0 | 1 | 1 | 0 |
| 35 | 0 | 1 | 16 | 1 | 0 | 1 | 1 | 1 |
| 56 | 0 | 5 | 9 | 0 | 0 | 0 | 0 | 1 |
| 61 | 0 | 10 | 15 | 0 | 0 | 1 | 1 | 1 |
| 40 | 0 | 10 | 7 | 0 | 1 | 1 | 2 | 0 |
| 64 | 0 | 5 | 11 | 1 | 0 | 1 | 1 | 0 |
| 48 | 1 | 10 | 9 | 0 | 0 | 1 | 1 | 1 |
| 53 | 0 | 10 | 13 | 1 | 0 | 0 | 0 | 1 |
| 50 | 0 | 7 | 7 | 1 | 0 | 1 | 1 | 0 |
| 58 | 0 | 10 | 9 | 0 | 0 | 1 | 1 | 0 |
| 61 | 0 | 10 | 11 | 0 | 0 | 1 | 1 | 1 |
| 49 | 0 | 7 | 13 | 0 | 0 | 1 | 1 | 1 |
| 54 | 0 | 10 | 9 | 1 | 0 | 1 | 1 | 1 |
| 51 | 1 | 10 | 13 | 0 | 0 | 1 | 1 | 1 |
| 63 | 1 | 10 | 5 | 1 | 0 | 1 | 1 | 0 |
| 57 | 1 | 10 | 14 | 1 | 0 | 0 | 0 | 1 |
| 66 | 0 | 10 | 9 | 0 | 1 | 0 | 1 | 0 |
| 46 | 1 | 10 | 11 | 1 | 0 | 1 | 1 | 0 |
| 61 | 0 | 10 | 11 | 0 | 0 | 1 | 1 | 0 |
| 56 | 1 | 10 | 9 | 0 | 0 | 1 | 1 | 0 |
| 66 | 0 | 5 | 11 | 0 | 0 | 1 | 1 | 1 |
| 54 | 0 | 10 |  | 0 | 0 | 1 | 1 | 0 |
| 48 | 0 | 5 | 24 | 0 | 0 | 1 | 1 | 1 |
| 52 | 0 | 1 | 10 | 0 | 0 | 1 | 1 | 0 |
| 58 | 0 | 10 | 13 | 0 | 0 | 1 | 1 | 1 |
| 56 | 0 | 10 | 10 | 0 | 0 | 1 | 1 | 0 |
| 46 | 0 | 5 | 18 | 0 | 0 | 1 | 1 | 0 |
| 53 | 0 | 5 | 11 | 0 | 0 | 1 | 1 | 1 |
| 54 | 1 | 10 | 11 | 0 | 0 | 1 | 1 | 1 |
| 68 | 0 | 10 | 10 | 0 | 0 | 1 | 1 | 1 |
| 69 | 0 | 10 | 11 | 0 | 0 | 1 | 1 | 1 |
| 57 | 0 | 1 | 11 | 0 | 0 | 1 | 1 | 1 |
| 34 | 0 | 10 | 11 | 0 | 0 | 1 | 1 | 0 |
| 48 | 0 | 5 | 13 | 0 | 0 | 1 | 1 | 0 |
| 65 | 0 | 11 | 7 | 0 | 0 | 1 | 1 | 0 |
| 55 | 0 | 10 | 10 | 0 | 0 | 1 | 1 | 1 |
| 65 | 0 | 1 | 7 | 0 | 0 | 1 | 1 | 0 |
| 46 | 1 | 10 | 9 | 0 | 0 | 1 | 1 | 1 |
| 69 | 0 | 10 | 9 | 1 | 0 | 1 | 1 | 0 |
| 63 | 0 | 5 | 11 | 0 | 0 | 1 | 1 | 0 |
| 61 | 0 | 10 | 11 | 0 | 1 | 0 | 1 | 0 |
| 62 | 0 | 10 | 10 | 1 | 0 | 1 | 1 | 1 |
| 61 | 0 | 1 | 9 | 0 | 1 | 0 | 1 | 1 |
| 63 | 0 | 10 | 11 | 0 | 0 | 1 | 1 | 0 |
| 60 | 0 | 10 | 19 | 0 | 1 | 1 | 2 | 1 |
| 61 | 0 | 10 | 9 | 0 | 0 | 1 | 1 | 0 |
| 60 | 0 | 5 | 19 | 1 | 0 | 1 | 1 | 1 |
| 56 | 0 | 5 | 11 | 0 | 0 | 1 | 1 | 0 |
| 68 | 0 | 10 | 10 | 0 | 0 | 1 | 1 | 0 |
| 64 | 0 | 19 | 5 | 0 | 0 | 0 | 0 | 1 |
| 44 | 1 | 10 | 13 | 0 | 0 | 1 | 1 | 0 |
| 64 | 0 | 10 | 18 | 1 | 1 | 1 | 2 | 0 |
| 35 | 0 | 10 | 20 | 0 | 0 | 1 | 1 | 1 |
| 74 | 0 | 10 | 15 | 0 | 0 | 1 | 1 | 0 |
| 46 | 0 | 10 | 11 | 0 | 0 | 1 | 1 | 0 |
| 72 | 0 | 10 | 7 | 0 | 0 | 1 | 1 | 0 |
| 54 | 0 | 8 | 7 | 0 | 1 | 1 | 2 | 0 |
| 48 | 0 | 10 | 11 | 0 | 1 | 0 | 1 | 1 |
| 55 | 0 | 10 | 11 | 0 | 0 | 1 | 1 | 0 |
| 59 | 0 | 10 | 11 | 0 | 1 | 0 | 1 | 1 |
| 59 | 0 | 1 | 7 | 0 | 0 | 1 | 1 | 1 |
| 63 | 1 | 10 | 9 | 1 | 0 | 1 | 1 | 1 |
| 50 | 1 | 7 | 7 | 0 | 0 | 0 | 0 | 1 |
| 41 | 0 | 1 | 13 | 0 | 0 | 1 | 1 | 0 |
| 69 | 1 | 10 | 7 | 0 | 0 | 1 | 1 | 0 |
| 71 | 0 | 10 | 11 | 0 | 1 | 0 | 1 | 0 |
| 64 | 0 | 7 | 6 | 0 | 0 | 1 | 1 | 0 |
| 59 | 0 | 7 | 7 | 0 | 1 | 1 | 2 | 0 |
| 65 | 1 | 10 | 13 | 1 | 0 | 1 | 1 | 1 |
| 67 | 0 | 10 | 13 | 0 | 0 | 1 | 1 | 0 |
| 47 | 1 | 10 | 13 | 0 | 0 | 1 | 1 | 1 |
| 66 | 0 | 10 | 11 | 0 | 0 | 1 | 1 | 0 |
| 56 | 0 | 10 | 11 | 0 | 0 | 1 | 1 | 0 |
| 68 | 0 | 10 | 30 | 1 | 0 | 1 | 1 | 1 |
| 87 | 0 | 10 | 6 | 0 | 0 | 1 | 1 | 1 |
| 65 | 0 | 7 | 7 | 0 | 1 | 1 | 2 | 0 |
| 64 | 0 | 10 | 10 | 0 | 0 | 1 | 1 | 1 |
| 69 | 1 | 10 | 10 | 0 | 0 | 1 | 1 | 1 |
| 68 | 0 | 10 | 11 | 0 | 1 | 0 | 1 | 0 |
| 60 | 0 | 7 | 7 | 1 | 0 | 1 | 1 | 1 |
| 65 | 0 | 10 | 9 | 1 | 0 | 0 | 0 | 1 |
| 62 | 0 | 10 | 12 | 0 | 0 | 0 | 0 | 1 |
| 55 | 0 | 10 | 9 | 0 | 0 | 0 | 0 | 0 |
| 66 | 0 | 10 | 9 | 1 | 0 | 1 | 1 | 1 |
| 61 | 1 | 10 | 12 | 0 | 0 | 1 | 1 | 0 |
| 59 | 0 | 5 | 11 | 0 | 0 | 1 | 1 | 0 |
| 62 | 0 | 10 | 9 | 1 | 0 | 1 | 1 | 1 |
| 45 | 0 | 7 | 11 | 0 | 0 | 1 | 1 | 1 |
| 58 | 0 | 10 | 11 | 0 | 1 | 0 | 1 | 0 |
| 61 | 0 | 7 | 7 | 0 | 0 | 1 | 1 | 0 |
| 61 | 0 | 10 | 9 | 0 | 0 | 1 | 1 | 0 |
| 62 | 0 | 10 | 9 | 1 | 1 | 0 | 1 | 0 |
| 73 | 0 | 5 | 11 | 0 | 0 | 1 | 1 | 1 |
| 69 | 0 | 10 | 7 | 0 | 0 | 1 | 1 | 0 |
| 45 | 0 | 10 | 13 | 1 | 0 | 1 | 1 | 0 |
| 60 | 0 | 10 | 22 | 1 | 0 | 1 | 1 | 1 |
| 56 | 0 | 10 | 9 | 0 | 0 | 1 | 1 | 1 |
| 66 | 0 | 10 | 9 | 0 | 0 | 1 | 1 | 0 |
| 42 | 1 | 10 | 9 | 0 | 0 | 1 | 1 | 0 |
| 55 | 0 | 1 | 11 | 1 | 0 | 1 | 1 | 0 |
| 73 | 1 | 10 | 9 | 1 | 0 | 1 | 1 | 1 |
| 38 | 0 | 1 | 11 | 0 | 0 | 0 | 0 | 0 |
| 59 | 0 | 19 | 7 | 0 | 0 | 1 | 1 | 1 |
| 63 | 0 | 7 | 8 | 0 | 0 | 1 | 1 | 0 |
| 60 | 0 | 10 | 11 | 1 | 0 | 1 | 1 | 1 |
| 61 | 0 | 1 | 7 | 0 | 0 | 1 | 1 | 0 |
| 64 | 0 | 10 | 7 | 1 | 0 | 1 | 1 | 0 |
| 72 | 1 | 10 | 9 | 0 | 0 | 1 | 1 | 1 |
| 62 | 1 | 10 | 11 | 1 | 0 | 1 | 1 | 0 |
| 59 | 0 | 7 | 7 | 0 | 0 | 1 | 1 | 0 |
| 75 | 1 | 7 | 11 | 1 | 0 | 1 | 1 | 1 |
| 42 | 0 | 10 | 11 | 1 | 0 | 1 | 1 | 0 |
| 67 | 0 | 10 | 11 | 0 | 0 | 1 | 1 | 0 |
| 66 | 0 | 1 | 7 | 0 | 0 | 1 | 1 | 1 |
| 55 | 0 | 1 | 7 | 0 | 0 | 1 | 1 | 1 |
| 51 | 1 | 5 | 9 | 0 | 0 | 1 | 1 | 0 |
| 77 | 0 | 1 | 11 | 1 | 0 | 1 | 1 | 1 |
| 62 | 0 | 1 | 15 | 0 | 0 | 1 | 1 | 1 |
| 58 | 0 | 10 | 10 | 1 | 0 | 1 | 1 | 1 |
| 66 | 0 | 5 | 6 | 1 | 0 | 1 | 1 | 0 |
| 62 | 0 | 10 | 7 | 0 | 0 | 1 | 1 | 1 |
| 68 | 0 | 5 | 9 | 0 | 0 | 1 | 1 | 0 |
| 66 | 0 | 10 | 15 | 0 | 0 | 1 | 1 | 1 |
| 71 | 1 | 10 | 11 | 0 | 1 | 0 | 1 | 0 |
| 64 | 0 | 10 | 10 | 1 | 0 | 1 | 1 | 1 |
| 50 | 0 | 1 | 10 | 0 | 1 | 0 | 1 | 1 |
| 54 | 0 | 1 | 7 | 1 | 0 | 1 | 1 | 1 |
| 44 | 0 | 10 | 9 | 0 | 0 | 1 | 1 | 1 |
| 44 | 0 | 10 | 13 | 0 | 0 | 1 | 1 | 1 |
| 69 | 0 | 1 | 8 | 0 | 0 | 1 | 1 | 0 |
| 67 | 0 | 10 | 8 | 1 | 0 | 1 | 1 | 1 |
| 45 | 0 | 10 | 11 | 1 | 0 | 1 | 1 | 0 |
| 65 | 0 | 7 | 9 | 1 | 0 | 1 | 1 | 0 |
| 55 | 0 | 10 | 14 | 0 | 0 | 1 | 1 | 0 |
| 53 | 0 | 1 | 9 | 1 | 0 | 1 | 1 | 0 |
| 49 | 0 | 10 | 16 | 0 | 0 | 1 | 1 | 0 |
| 51 | 0 | 7 | 11 | 1 | 0 | 0 | 0 | 0 |
| 47 | 0 | 10 | 10 | 1 | 0 | 1 | 1 | 0 |
| 71 | 0 | 10 | 5 | 0 | 1 | 0 | 1 | 1 |
| 45 | 0 | 10 | 13 | 1 | 0 | 1 | 1 | 1 |
| 48 | 0 | 1 | 9 | 1 | 1 | 1 | 2 | 1 |
| 58 | 0 | 10 | 11 | 1 | 0 | 1 | 1 | 1 |
| 62 | 0 | 10 | 11 | 1 | 1 | 0 | 1 | 1 |
| 63 | 0 | 12 | 11 | 1 | 0 | 1 | 1 | 1 |
| 53 | 0 | 10 | 13 | 0 | 0 | 1 | 1 | 0 |
| 70 | 0 | 10 | 19 | 0 | 0 | 0 | 0 | 1 |
| 69 | 0 | 10 | 11 | 0 | 1 | 1 | 2 | 1 |
| 67 | 0 | 10 | 7 | 0 | 0 | 1 | 1 | 0 |
| 61 | 0 | 10 | 11 | 1 | 0 | 1 | 1 | 1 |
| 68 | 0 | 10 | 11 | 1 | 0 | 1 | 1 | 0 |
| 62 | 0 | 7 | 7 | 0 | 0 | 1 | 1 | 0 |
| 60 | 0 | 1 | 9 | 0 | 0 | 1 | 1 | 0 |
| 61 | 0 | 10 | 13 | 0 | 0 | 0 | 0 | 0 |
| 61 | 0 | 10 | 9 | 0 | 0 | 1 | 1 | 1 |
| 52 | 0 | 7 | 11 | 0 | 0 | 1 | 1 | 1 |
| 47 | 0 | 1 | 17 | 1 | 0 | 1 | 1 | 0 |
| 54 | 0 | 5 | 13 | 1 | 1 | 0 | 1 | 0 |
| 78 | 0 | 10 | 10 | 0 | 0 | 1 | 1 | 1 |
| 70 | 0 | 7 | 11 | 1 | 0 | 1 | 1 | 0 |
| 38 | 0 | 5 | 14 | 0 | 0 | 1 | 1 | 1 |
| 50 | 0 | 11 | 7 | 1 | 0 | 1 | 1 | 0 |
| 45 | 0 | 10 | 11 | 0 | 0 | 1 | 1 | 0 |
| 51 | 0 | 10 | 17 | 1 | 0 | 1 | 1 | 0 |
| 41 | 0 | 1 | 11 | 1 | 1 | 1 | 2 | 0 |
| 68 | 1 | 10 | 11 | 0 | 0 | 1 | 1 | 1 |
| 55 | 0 | 10 | 11 | 0 | 0 | 1 | 1 | 0 |
| 55 | 0 | 10 | 15 | 0 | 0 | 1 | 1 | 1 |
| 59 | 0 | 10 | 21 | 1 | 1 | 0 | 1 | 1 |
| 52 | 0 | 5 | 8 | 0 | 0 | 1 | 1 | 1 |
| 59 | 0 | 7 | 8 | 0 | 0 | 1 | 1 | 1 |
| 66 | 0 | 10 | 9 | 1 | 1 | 0 | 1 | 1 |
| 66 | 0 | 1 | 11 | 0 | 1 | 0 | 1 | 1 |
| 47 | 0 | 10 | 11 | 1 | 0 | 1 | 1 | 1 |
| 59 | 0 | 10 | 17 | 1 | 0 | 1 | 1 | 1 |
| 54 | 0 | 5 | 9 | 1 | 0 | 1 | 1 | 0 |
| 66 | 0 | 10 | 9 | 1 | 0 | 1 | 1 | 1 |
| 66 | 0 | 10 | 11 | 0 | 0 | 1 | 1 | 0 |
| 68 | 0 | 10 | 13 | 1 | 0 | 1 | 1 | 1 |
| 49 | 1 | 10 | 13 | 0 | 0 | 1 | 1 | 1 |
| 48 | 1 | 10 | 15 | 1 | 0 | 1 | 1 | 1 |
| 48 | 0 | 10 | 11 | 1 | 0 | 1 | 1 | 0 |
| 70 | 0 | 1 | 13 | 1 | 0 | 1 | 1 | 0 |
| 62 | 1 | 1 | 10 | 0 | 0 | 0 | 0 | 0 |
| 55 | 0 | 10 | 10 | 1 | 0 | 1 | 1 | 1 |
| 58 | 0 | 7 | 9 | 1 | 0 | 1 | 1 | 1 |
| 55 | 0 | 10 | 11 | 0 | 0 | 0 | 0 |  |
| 55 | 0 | 10 | 13 | 1 | 0 | 1 | 1 | 0 |
| 39 | 1 | 1 | 14 | 1 | 0 | 1 | 1 | 0 |
| 58 | 0 | 10 | 11 | 1 | 1 | 1 | 2 | 1 |
| 66 | 0 | 5 | 8 | 0 | 1 | 1 | 2 | 1 |
| 62 | 0 | 10 | 10 | 1 | 0 | 1 | 1 | 1 |
| 67 | 0 | 10 | 11 | 0 | 1 | 0 | 1 | 0 |
| 60 | 0 | 10 | 10 | 0 | 0 | 1 | 1 | 0 |
| 62 | 0 | 10 | 11 | 0 | 1 | 1 | 2 | 0 |
| 55 | 0 | 10 | 12 | 1 | 1 | 1 | 2 | 0 |
| 51 | 0 | 10 | 11 | 1 | 0 | 1 | 1 | 1 |
| 64 | 0 | 10 | 9 | 1 | 1 | 1 | 2 | 1 |
| 58 | 0 | 5 | 9 | 0 | 0 | 1 | 1 | 1 |
| 74 | 0 | 5 | 10 | 0 | 0 | 1 | 1 | 0 |
| 61 | 0 | 5 | 11 | 1 | 0 | 1 | 1 | 0 |
| 50 | 0 | 7 | 11 | 0 | 0 | 1 | 1 | 1 |
| 63 | 0 | 10 | 13 | 0 | 1 | 0 | 1 | 0 |
| 56 | 0 | 10 | 13 | 0 | 0 | 1 | 1 | 0 |
